# Diagnose This quiz

**Published:** 2014

**Figure:**
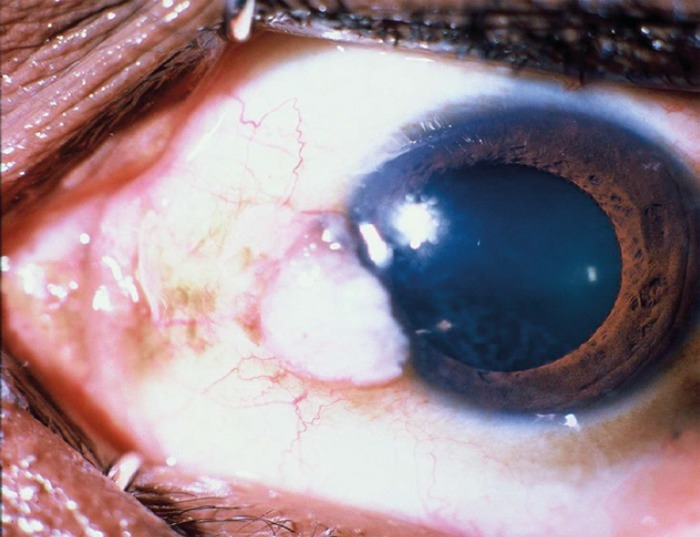


This 40-year-old patient in Africa presented with a history of a non-painful swelling on the conjunctiva first noticed three months ago. There was no history of injury. The other eye was normal.

1. What is the diagnosis?
  □ **a.** Ptyergium
  □ **b.** Bitot's spots
  □ **c.** Episcleritis
  □ **d.** Squamous cell carcinoma
  □ **e.** Pinguecula
2. Which of the following are known risk factors for the answer to question 1? Select all that apply.
  □ **a.** Iritis
  □ **b.** HIV infection
  □ **c.** Ultraviolet radiation
  □ **d.** Bowen's disease
  □ **e.** Pinguecula
3. Which of the following is the first line recommended treatment for the answer to question 1?
  □ **a.** Chemotherapy
  □ **b.** Laser treatment
  □ **c.** Irradiation
  □ **d.** Topical steroids
  □ **e.** Wide surgical excision + cryotherapy

## ANSWERS

1. Diagnosis: d. Squamous cell carcinoma.
2. Risk factors: b, c and d are all true. HIV positive status, exposure to ultraviolet light radiation, and the pre- (early) cancerous condition, Bowen's disease, may all predispose to squamous cell carcinoma of the conjunctiva.
3. Recommended treatment: Wide (2 mm edges) surgical excision leaving the sclera bare. If possible, cryotherapy should be applied to the bare sclera and conjunctival margins.

